# Reply to: Biogeographic implications of plant stature and microclimate in cold regions

**DOI:** 10.1038/s42003-023-05033-4

**Published:** 2023-06-26

**Authors:** Ulf Büntgen, Alma Piermattei, Jiri Dolezal, Paul Dupree, Alan Crivellaro

**Affiliations:** 1 Department of Geography, University of Cambridge, CB2 3EN Cambridge, UK; 2 Swiss Federal Research Institute WSL, 8903 Birmensdorf, Switzerland; 3 Global Change Research Institute CAS, 603 00 Brno, Czech Republic; 4 Department of Geography, Faculty of Science Masaryk University, 611 37 Brno, Czech Republic; 5 Department of Agricultural, Forest and Food Sciences, University of Torino, Largo Paolo Braccini, 2, 10095 Grugliasco, TO Italy; 6 Forest Biometrics Laboratory, Faculty of Forestry, Stefan cel Mare University of Suceava, 720229 Suceava, Romania; 7 Institute of Botany, Academy of Sciences of the Czech Republic, 379 01 Trebon, Czech Republic; 8 Department of Botany, Faculty of Science University of South Bohemia, 370 05 Ceske Budejovice, Czech Republic; 9 Department of Biochemistry, University of Cambridge, CB2 1QW Cambridge, UK

**Keywords:** Plant ecology, Biogeography

**replying to** C. Körner et al. *Communications Biology* 10.1038/s42003-023-05032-5 (2023)

Pondering on the essence of good science, we are delighted that our initial, wood anatomical work^1^ on the biogeographic implication of a probable temperature-induced constrain on plant cell wall lignification not only stimulates conceptual discussion but also provokes relational thinking. We further consider scrutinizing previous publications a fundamental element of innovative research rather than a criterion for suspicion. Here, we critically reflect on the available evidence for a reduced ability of plants to lignify their secondary cell walls under extremely cold growing season temperatures.

Foremost, we acknowledge critical scientific contributions to better understand the life of Alpine plants^2^ and the global position of Alpine treelines^3^. We also understand the challenges associated with upscaling cell-level information from individual plants to describe and explain large-scale biogeographic phenomena, such as the global treeline. However, the potential for new empirical data and innovative conceptual thinking to elucidate patterns otherwise attributed to misleading factors is an integral part of scientific progress.

The current Matters Arising by C. Körner et al. seems motivated by methodological and conceptual misunderstanding that distracts from the main topic under debate, because it is a decreasing trend of cell wall lignification towards colder environments that matters above all^1,4^. Small plants growing above the treeline may contain less lignin than tall, upright-growing plants at lower elevations, because extremely cold temperatures are hypothesised to affect the lignification process of secondary cell walls. However, the upright stems of tall plants are (by definition) always lignified (see details below). We therefore suggest revisiting the existing theories about a thermal distribution limit of upright plant growth, i.e., the lifeform tree, and considering a range of biochemical and biomechanical factors in future treeline research, empirically and conceptually.

Reflecting on the data used, methods applied and results obtained^1^, we refute the five main issues raised by C. Körner et al.: (1) In situ, high-resolution meteorological surface air and particularly soil temperature measurements unfortunately do not exist for each individual site from where plant samples were collected or extrapolated. Nevertheless, we consider this theoretical limitation to be irrelevant for the ground-based detection and interpretation of large-scale biogeographic patterns, as previously done for the global treeline position. While high surface temperatures can be reached well beyond the alpine and arctic treelines, the spatiotemporally constrained example shown by C. Körner et al. is irrelevant, because cold conditions define species’ upper and poleward distribution ranges, and the infra-red thermal map would likely be inverse during cloudy days and night-time recordings. (2) Associated with some degree of uncertainty, the available WorldClim data at 2.5-minute spatial resolution allow macro ecological trends to be identified. This is encouraging as the global treeline position has been described by a universal temperature threshold rather than varying microclimatic parameters, and the realised and potential treeline, i.e., the position of treeline trees and the thermal treeline isotherm, are almost never in equilibrium. (3) There is little anatomical, morphological and physiological support for a classical lifeform separation^5–7^. Herbs smaller than circa 10 cm vertical height tend to exhibit reduced cell wall lignification at colder sites, as well as higher elevations and latitudes, whereas the xylem of upright growing shrubs and trees must be lignified because they would not exist otherwise. (4) Anatomical thin sections were exclusively taken from the outermost mature and physiologically active stem tissues at root collar, excluding disturbances, dead and developing cells, as well as the cambium. Though not ideal, we expect the amount of parenchyma cells to be neglectable in the overall picture. Plant stems with less lignified secondary cell walls have reduced wood mechanical strength and a limited capacity for long-distance and upright water transport. The separation between ‘wood’, ‘woody’ and ‘woodiness’ requires anatomical, morphological and physiological specification^5^, but all plants with a secondary cambium that produces xylem cells should be considered as ‘woody’ species, including *Arabidopsis*^8^. (5) Since different arguments have been exchanged^1,4^, and the coldest place on Earth with plants seems warmer than initially proposed^9^, the ongoing debate is likely to continue stimulating research at the extremes of both Arctic and alpine plant growth. For instance, an in situ cooling experiment performed throughout the 2009 growing season at the upper treeline in the Swiss Alps suggests a thermal threshold below which the lignification process in Stone pines (*Pinus uncinata*) was interrupted^10^, and a layer of ‘blue’ cell walls became visible after double-staining. This finding corroborates recent evidence for exceptional ‘Blue Ring’ occurrence in Estonian Scots pines (*Pinus sylvestris*) due to remarkably low temperatures towards the end of the 1976 growing season^11^.

In synthesis, we concur that yet outstanding questions in treeline research should guide future investigations. We also agree that the remaining lack of mechanistic understanding of how extreme temperatures exactly affect metabolic processes in trees at their cold distribution limit calls for advanced treeline studies across different spatiotemporal scales^12^. There is no doubt that treeline studies including timely aspects of macro ecology and plant physiology will gain in importance under global climate change. Understanding the causes and consequences of recent ‘Arctic Greening’, for instance, is just one biogeographic example in which a more nuanced eco-physiological perspective on cell wall biochemistry would be helpful. We also accept the importance of microclimate and different forms of lignin for xylogenesis and therefore recommend well-replicated plant stem anatomical analyses of different cell functional types in small herbs and large trees within and between species. As pointed out by C. Körner et al., not only our assessment of the ‘Schweingruber’ collection^1^, but also any other large-scale wood anatomical examination of the degree of stem cell wall lignification in plants would benefit from the inclusion of gymnosperms. Though, the arguments by C. Körner et al. that not enough trees were included, that trees from treeline sites were missing, and that trees did not show trends in cell wall lignification are simply invalid because the stems of upright, tall trees are either lignified or they do not exist.

Although we are not yet able to explain the reasons for reduced cell wall lignification in small plants growing under relatively warm conditions (further analyses and experimentation are needed), we do recognise mounting evidence for a cold temperature-induced reduction in cell wall lignification based on local and historical evidence^13,14^ (Fig. 1), which complements the evidence from our global scale study^1^. Finally, we emphasise two questions to provoke critical thinking and empirical exploration: Why are secondary cell walls in the stems of small plants growing under extreme cold conditions often less lignified compared to samples of the same species from warmer sites? Why are ‘Blue Rings’ often found after large volcanic eruptions when growing seasons are interrupted by ephemeral cold spells?

**Fig. 1:**
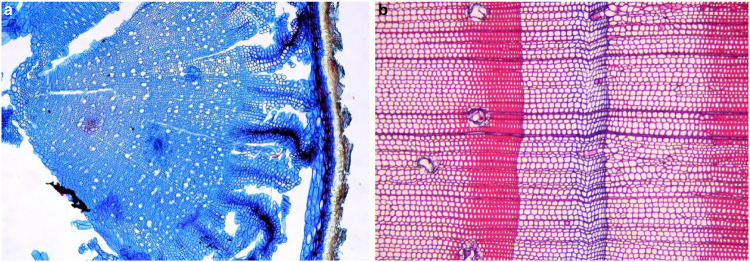
Independent lines of ‘Blue’ evidence for cooling-induced reduction in cell wall lignification of plant stems. **a** Local evidence from a small alpine herb (*Ladakiella klimesii*) that was collected at circa 6150 m asl in the Himalayas^13^ (image reproduced from ref. ^13^). There is almost no lignin visible in any of the 20 annual growth rings. **b** Historical evidence from a relict *Pinus sylvestris* tree that grew in northern Scandinavia^15^, which formed a so-called ‘Blue Ring’ during the coldest summer of the past 2000 years and now offers a time-for-space surrogate in wood anatomical and biogeographic research (image reproduced from ref. ^15^).

## References

[1] Crivellaro A, Piermattei A, Dolezal J, Dupree P, Büntgen U (2022). Biogeographic implication of temperature-induced plant cell wall lignification. Comm. Biol..

[2] Körner, C. Alpine Plant Life. *Functional Plant Ecology of High Mountain Ecosystems* (Springer International Publishing, 2021).

[3] Körner, C. Alpine Treelines. *Functional Ecology of the Global High Elevation Tree Limits* (Springer Basel, 2012).

[4] Crivellaro A, Büntgen U (2020). New evidence of thermally-constraint plant cell wall lignification. Trends Plant Sci..

[5] Schweingruber FH, Büntgen U (2013). What is ‘wood’ – an anatomical re-definition. Dendrochronologia.

[6] Büntgen U, Psomas A, Schweingruber FH (2014). Introducing wood anatomical and dendrochronological aspects of herbaceous plants: applications of the Xylem Database to vegetation science. J. Veg. Sci..

[7] Schweingruber FH (2013). Evaluating the wood anatomical and dendroecological potential of Arctic dwarf shrubs. IAWA J..

[8] Zhang, J., Elo, A. & Helariutta, Y. *Arabidopsis* as a model for wood formation. *Curr. Opinion Biotechnol*. **22**, 293–299 (2011). 10.1016/j.copbio.2010.11.008 21144727

[9] Körner C (2019). Life at 0 °C: the biology of the alpine snowbed plant *Soldanella pulsatilla*. Alp. Bot..

[10] Körner C, Lenz A, Hoch G (2023). Chronic in situ tissue cooling does not reduce lignification at the Swiss treeline but enhances the risk of ‘blue’ frost rings. Alp. Bot..

[11] Greaves C (2023). Remarkably high blue ring occurrence in Estonian Scots pines in 1976 reveals wood anatomical evidence of extreme autumnal cooling. Trees.

[12] Büntgen U (2022). Common Era treeline fluctuations and their implications for climate reconstructions. Glob. Plan. Change.

[13] Dolezal J (2016). Vegetation dynamics at the upper elevational limit of vascular plants in Himalaya. Sci. Rep..

[14] Piermattei A (2020). A millennium-long ‘Blue-Ring’ chronology from the Spanish Pyrenees reveals sever ephemeral summer cooling after volcanic eruptions. Environ. Res. Lett..

[15] Büntgen U (2022). Global wood anatomical network reveals repeated summer cooling at the onset of the Late Antique Little Ice Age. Sci. Bull..

